# Development of a blood-based lipidomic fat quality score for the risk of ischemic stroke

**DOI:** 10.1093/esj/23969873251367250

**Published:** 2026-01-01

**Authors:** Iolanda Lázaro, Leila Luján-Barroso, Natalia Soldevila-Domenech, Antonio J Amor, Emilio Ortega, Emilio Ros, Maria-José Sánchez, Miguel Rodríguez-Barranco, Marcela Guevara, Conchi Moreno-Iribas, Helmut Schröder, Montserrat Fitó, Nathan L Tintle, Nathan Ryder, William S Harris, Antonio Agudo, Aleix Sala-Vila

**Affiliations:** Hospital del Mar Medical Research Institute, Barcelona, Spain; Barcelonaβeta Brain Research Center (BBRC), Pasqual Maragall Foundation, Barcelona, Spain; Centro de Investigación Biomédica en Red de la Fisiopatología de la Obesidad y Nutrición (CIBEROBN), Instituto de Salud Carlos III, Madrid, Spain; Unit of Nutrition and Cancer, Catalan Institute of Oncology - ICO, L’Hospitalet de Llobregat, Spain; Nutrition and Cancer Group; Epidemiology, Public Health, Cancer Prevention and Palliative Care Program; Bellvitge Biomedical Research Institute - IDIBELL, L’Hospitalet de Llobregat, Spain; Hospital del Mar Medical Research Institute, Barcelona, Spain; Endocrinology and Nutrition Department, Hospital Clínic de Barcelona, Spain; Centro de Investigación Biomédica en Red de la Fisiopatología de la Obesidad y Nutrición (CIBEROBN), Instituto de Salud Carlos III, Madrid, Spain; Endocrinology and Nutrition Department, Hospital Clínic de Barcelona, Spain; Translational Research in Diabetes, Lipids and Obesity, Institut d’Investigacions Biomèdiques August Pi i Sunyer (IDIBAPS), Barcelona, Spain; Centro de Investigación Biomédica en Red de la Fisiopatología de la Obesidad y Nutrición (CIBEROBN), Instituto de Salud Carlos III, Madrid, Spain; Cardiovascular Risk, Nutrition and Aging (IDIBAPS), Barcelona, Spain; Escuela Andaluza de Salud Pública (EASP), Granada, Spain; Instituto de Investigación Biosanitaria ibs.GRANADA, Granada, Spain; Centro de Investigación Biomédica en Red de Epidemiología y Salud Pública (CIBERESP), Madrid, Spain; Escuela Andaluza de Salud Pública (EASP), Granada, Spain; Instituto de Investigación Biosanitaria ibs.GRANADA, Granada, Spain; Centro de Investigación Biomédica en Red de Epidemiología y Salud Pública (CIBERESP), Madrid, Spain; Department of Epidemiology, Murcia Region Health Council-IMIB Arrixaca, Murcia, Spain; Centro de Investigación Biomédica en Red de Epidemiología y Salud Pública (CIBERESP), Madrid, Spain; Instituto de Salud Pública de Navarra, Pamplona, Spain; Navarra Institute for Health Research (IdiSNA), Pamplona, Spain; Centro de Investigación Biomédica en Red de Epidemiología y Salud Pública (CIBERESP), Madrid, Spain; Instituto de Salud Pública de Navarra, Pamplona, Spain; Navarra Institute for Health Research (IdiSNA), Pamplona, Spain; Unit of Nutrition and Cancer, Catalan Institute of Oncology - ICO, L’Hospitalet de Llobregat, Spain; Nutrition and Cancer Group; Epidemiology, Public Health, Cancer Prevention and Palliative Care Program; Bellvitge Biomedical Research Institute - IDIBELL, L’Hospitalet de Llobregat, Spain; Hospital del Mar Medical Research Institute, Barcelona, Spain; Centro de Investigación Biomédica en Red de Epidemiología y Salud Pública (CIBERESP), Madrid, Spain; Hospital del Mar Medical Research Institute, Barcelona, Spain; Centro de Investigación Biomédica en Red de la Fisiopatología de la Obesidad y Nutrición (CIBEROBN), Instituto de Salud Carlos III, Madrid, Spain; Department of Population Health Nursing Science, College of Nursing, University of Illinois-Chicago, Chicago, IL, USA; Fatty Acid Research Institute, Sioux Falls, SD, USA; Fatty Acid Research Institute, Sioux Falls, SD, USA; Fatty Acid Research Institute, Sioux Falls, SD, USA; Sanford School of Medicine, University of South Dakota, Sioux Falls, SD, USA; Unit of Nutrition and Cancer, Catalan Institute of Oncology - ICO, L’Hospitalet de Llobregat, Spain; Nutrition and Cancer Group; Epidemiology, Public Health, Cancer Prevention and Palliative Care Program; Bellvitge Biomedical Research Institute - IDIBELL, L’Hospitalet de Llobregat, Spain; Hospital del Mar Medical Research Institute, Barcelona, Spain; Barcelonaβeta Brain Research Center (BBRC), Pasqual Maragall Foundation, Barcelona, Spain; Centro de Investigación Biomédica en Red de la Fisiopatología de la Obesidad y Nutrición (CIBEROBN), Instituto de Salud Carlos III, Madrid, Spain; Fatty Acid Research Institute, Sioux Falls, SD, USA

**Keywords:** Biomarkers, fatty acids, diet, nutrition, lifestyle

## Abstract

**Introduction:**

Poor-quality diets promote ischemic stroke. Red blood cell fatty acids (RBC-FAs) are objective, long-term biomarkers of diet. In a case-control study nested in the European Prospective Investigation into Cancer and Nutrition (EPIC)-Spain, we developed a blood-based lipidomic fat quality (LFQ) score considering pre-defined RBC-FA diet-related biomarkers, and examined whether LFQ score relates to the risk of ischemic stroke.

**Patients and methods:**

We determined the RBC-FAs (*n* = 438 cases of incident ischemic stroke, *n* = 438 matched controls). For each participant, we scored 1 for each beneficial metric (C15:0+C17:0; C18:2n-6; C18:3n-3; C20:5n-3; C22:6n-3) ⩾the median of the control group; and 1 for each detrimental metric (C16:0; C16:1n-7; C18:0) <the median of the control group. LFQ score resulted from the 8-component sum (range = 0-8; higher values, higher fat quality). We explored the validity of findings in a different background (*n* = 2468 participants from the Framingham Offspring Study without ischemic stroke at baseline, 12-year median follow-up, *n* = 121 cases).

**Results:**

In a fully adjusted model, the Odds Ratio (OR) for ischemic stroke was 0.86 (95% confidence interval [CI] = 0.77–0.95) for each 1-unit increase of the LFQ score. Compared to individuals at the lowest category of LFQ score (0–3 points), those at the top category (5–8 points) had lower odds (OR = 0.64, 95% CI = 0.44–0.94). The findings were similar in the Framingham Offspring Study (Hazard Ratio [HR] for each 1-unit increase = 0.83; 95% CI = 0.70–0.99; HR for those at top category = 0.49; 95% CI = 0.29–0.84, compared to those at the lowest category).

**Conclusion:**

Low blood-based LFQ scores relate to a high risk of ischemic stroke.

## Graphical Abstract

**Graphical abstract:**
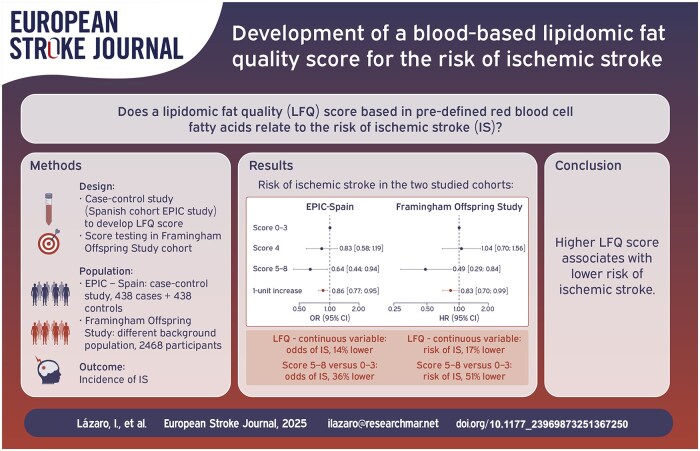

## Introduction

Ischemic stroke remains a leading cause of mortality and a major cause of morbidity and disability worldwide^1^ and the global burden is expected to increase.^2^ Besides control of cardiovascular risk factors, mainly hypertension, diabetes and dyslipidemia, the primary prevention of ischemic stroke is mostly based upon lifestyle modification, including non-smoking, physical exercise, and adherence to a healthy diet.^3^ Several indices have been recently developed to assess the overall quality (healthfulness) of the diet^4^ and better characterize nutritional status. While the strength of the association of diet quality indices with cardiovascular events in epidemiologic studies appears to be clinically important,^4^ major gaps in knowledge still exist. The use of self-reported data, inherent to obtaining indices of dietary quality, compromises accuracy when assessing food consumption and deriving nutrient intake. This might be partially circumvented by direct chemical measurement of nutrients and/or bioactives in blood (objective biomarkers of dietary intake).^5^

The long-standing link between dietary fatty acids (FAs) and cardiovascular disease^6^ prompted a large body of observational research on FA biomarkers of dietary intake and the risk of incident ischemic stroke. A few landmark papers pooled data from many cohorts, but they largely focused on selected, individual FA species. They include the essential FAs linoleic (C18:2n-6)^7^ and alpha-linolenic (C18:3n-3) acids^8^ (mostly supplied by nuts, oily seeds, and vegetable oils), and the long-chain n-3 FAs eicosapentaenoic (C20:5n-3) and docosahexaenoic (C22:6n-3) acids (mostly supplied by fatty fish).^9^ However, there are other FA biomarkers of dietary intake with theoretical/established effect on cardiovascular health, including of C15:0 and C17:0 (mostly supplied by dairy products); and palmitic (C16:0), palmitoleic (C16:1n-7), and stearic (C18:0) acids, which result from hepatic de novo lipogenesis [DNL], a metabolic process to limit excess of energy intake from dietary starch, sugar, protein, or alcohol).^10^

Rather than searching for individual associations for each exposure of interest, we here combined these nine metrics to develop a blood-based lipidomic fat quality (LFQ) score (higher scores indicating higher lipidomic fat quality), and further examined whether increasing LFQ score relates to a lower risk of ischemic stroke. We addressed this issue in a case-control study nested within the Spanish cohort of the European Prospective Investigation into Cancer and Nutrition (EPIC) study^11^ (*n* = 438 cases of ischemic stroke and 438 matched controls). Then, we explored whether the LFQ score related to incident ischemic stroke in a population with a different background, the Framingham Offspring Study cohort.^12^

## Methods

### Study populations

For this case-control study we included participants from three centers from EPIC-Spain cohort^11^ willing to participate (Granada, Murcia, and Navarra; *n* = 24,479 participants; aged 29–69 years at recruitment). At enrollment, which took place between October 1992 and July 1996, participants provided personal, clinical and dietary data in face-to-face interviews. We used questionnaires to obtain baseline information on sociodemographic and lifestyle factors^11^ including education, tobacco use, food consumption,^13^ physical activity,^14^ and medical history. Trained nurses measured weight and height. Fasting venous blood samples were drawn and immediately processed and were divided into 0.5 mL aliquots of plasma, serum, red blood cells (RBCs), and buffy coat, which were stored in liquid nitrogen tanks at −196°C for further analyses.

We examined whether the findings in EPIC-Spain were also observed in the Framingham Offspring Study cohort,^12^ originating in 1971 including children of the original Framingham Heart Study cohort^15^ and their spouses. The Offspring cohort originally consisted of 5124 participants who have now been studied over up to nine examination cycles, approximately once every 4 years. Of the original Framingham Offspring Study cohort, 3021 attended their eighth examination cycle (2005–2008), at which RBCs were isolated from blood drawn after a 10- to 12-h fast and frozen at −80°C immediately after collection on *n* = 2857 individuals. Simultaneous to RBC collection, participants were assessed on numerous clinical, dietary,^16^ lifestyle and adiposity variables of interest.

### Follow-up for incident ischemic stroke and vital status

We included all documented cases of definite fatal or non-fatal ischemic stroke in the three EPIC-Spain centers participating in this study. Participants who at recruitment had a prior diagnosis of stroke that was validated thereafter were excluded from further analyses. Incident stroke cases were identified by record linkage with hospital discharge databases (codes 430–438 of the International Classification of Diseases, Ninth Revision; Clinical Modification (ICD-9-CM)) and primary care records (codes K89, K90, and K92 from the International Classification of Primary Care and ICD-9 codes 430–438). Fatal cases were identified by record linkage with the National Institute of Statistics (INE) using ICD-9 codes 430–438 and ICD-10 codes I60–I69. Trained health professionals carried out a validation process to confirm and classify all identified stroke events by carefully reviewing hospital records or, if not available, records from primary-care physicians, and registering the date of diagnosis. Stroke cases were classified based on symptoms, presence of cerebrovascular risk factors and specific examinations (computerized tomography, magnetic resonance imaging, angiography, Doppler imaging and/or lumbar puncture) following the 2006 guidelines of the Spanish Society of Neurology^17^ for ischemic, hemorrhagic (cerebral and subarachnoid) or unspecific strokes. Two expert neurologists helped in the classification of the most difficult cases.

During the follow-up period (median, 17 years) 452 incident ischemic strokes were documented. For each case, we chose one matched control by using an incidence density sampling protocol,^18^ from appropriate risk sets consisting of cohort members alive and free of ischemic stroke at the time of diagnosis of the index case. Matching criteria were recruiting center, sex, age at enrollment (within ±2.5 years), and the date of blood collection (within ± 3 months). RBC samples were missing in four cases. Therefore, RBC FA analysis was conducted in *n* = 448 samples and *n* = 448 matched controls (*n* = 896). However, we observed technically unacceptable results in *n* = 10 samples, from either cases or controls. Therefore, we excluded these samples along with their matched ones, resulting in 438 pairs of cases and controls (*n* = 876) included in the statistical analyses (see flow-chart in Supplemental Figure 1).

In the Framingham Offspring Study cohort (longitudinal prospective cohort design instead of a case-control study nested in a prospective cohort), we considered ischemic strokes which occurred between examination 8 and December 31 2018. Stroke incidence was assessed through the continuous monitoring of hospital admissions in Framingham and by reviewing all available medical records. Ischemic stroke was defined as a focal neurological deficit of presumed vascular cause with a sudden onset and lasting for at least 24 h, in the absence of an intracranial hemorrhage (diagnosed by CT when available, or laboratory results, clinical information or autopsy findings when CT was not available) or other brain disorder that could cause focal neurological deficits. Ischemic stroke included atherosclerotic brain infarction, cerebral embolism from a documented cardiac source, and transient ischemic attack. Events were ascertained by ⩾2 neurologists via consensus. From the participants attending to the exam 8 with available data on RBC FA measurements (*n* = 2857), we excluded those with a history of stroke at exam 8 (*n* = 131), those who suffered from ischemic stroke <1 year from baseline (*n* = 34), and those missing body mass index, alcohol, or diet information (*n* = 224), leaving 2468 eligible for the present investigation. Follow-up ranged between 1.0 and 13.8 years, with a median of 12.1 years. During this time, a total of *n* = 121 cases of ischemic stroke were documented.

### Determination of RBC FAs

In EPIC-Spain, assays were conducted at Hospital del Mar Research Institute (Barcelona), using a method that had been previously validated against the reference method used to determine the RBC FA profile in the Framingham Offspring Study cohort.^19^ Forty microliters of RBCs were spiked with 10 μg of the internal standard 1,2-dinonadecanoyl-sn-glycero-3-phosphocholine (Avanti, Merck), hemolysed with distilled water and centrifuged. The supernatant (containing mainly hemoglobin) was discarded, and the pellet (consisting of RBC membranes) was directly trans-esterified to obtain FA methyl esters (FAMEs), as previously described.^19^ FAMEs were analyzed by gas chromatography/electron ionization mass spectrometry (GC/MSEI), using an Agilent 6890N GC equipped with an Agilent 7683 autosampler, and an Agilent 5973N mass spectrometry detector. FAMEs were separated with a J&W DB-FastFAME capillary column (30 m × 0.2 mm × 0.25 μm film thickness, Agilent). The injector temperature was set at 250°C, and 1 μL injections were performed (split ratio 25:1). GC was run using an optimized temperature program, as follows: the program started at 50°C, held for 0.5 min, increased to 194°C at a rate of 25°C/min, held for 1 min, increased to 245°C at a rate of 5°C/min, and held for 3 min. Helium was used as carrier gas (14 psi, constant pressure mode). FAMEs were detected using the selected ion monitoring mode. Several m/z ions common to saturated, monounsaturated, and polyunsaturated FAMEs were monitored.^20^ Twelve mixtures of FAME external calibration standards, spiked with C19:0-methyl ester in an equivalent amount to that included in samples as phospholipid, were prepared by diluting FAME mix certified reference material (Supelco 37 Component FAME Mix, Merck) in hexane. The concentrations of FAMEs in the samples were calculated by linear regression of the peak area ratio relative to that of the internal standard. The amount of each FA is expressed as the percentage of the total amount of 22 determined FAs (see detailed list in Supplemental Table 1).

In the Framingham Offspring Study cohort, RBC FAMEs were also generated by acid transesterification with boron trifluoride and analyzed by gas chromatography using a GC2010 Gas Chromatograph (Shimadzu Corporation, Columbia, MD) equipped with a SP2560, 100 m column (Supelco, Bellefonte, PA). Twenty-eight FAs were identified by comparison with a standard mixture of FAs characteristic of RBCs. After response factor correction, results were calculated as a percentage of total identified FAs.

### Score development

We used control participants from the EPIC-Spain cohort to develop the LFQ score. We included nine pre-defined metrics of (i) exogenous (dietary) FAs with absent or marginal endogenous synthesis, namely the sum of C15:0 and C17:0 (dairy products); C18:2n-6 and C18:3n-3 (nuts, oily seeds, and vegetable oils); eicosapentaenoic (C20:5n-3) and docosahexaenoic (C22:6n-3) acids (fatty fish); and (ii) FAs resulting from DNL, namely palmitic (C16:0), palmitoleic (C16:1n-7), and stearic (C18:0) acids. Although oleic acid (C18:1n-9 *cis*) is also an end-product of DNL, we decided against considering it for the LFQ score because in Mediterranean regions its blood status largely reflects consumption of olive oil (a well-known healthy fat),^21^ hence its enrichment in blood can be considered either beneficial (from olive oil) or detrimental (from DNL). Each of these pre-defined FA was selected on basis of a proved/theoretical effect on cardiovascular health. Beneficial FAs were C15:0 + C17:0,^22^ C18:2n-6,^7^ C18:3n-3,^8^ C20:5n-3,^9^ and C22:6n-3^9^; while detrimental ones were C16:0,^22^ C18:0,^23^ and C16:1n-7.^24^

In the EPIC-Spain cohort, individuals were assigned a score of 1 for each theoretical beneficial FA (C15:0+C17:0; C18:2n-6; C18:3n-3; C20:5n-3; C22:6n-3) if his or her RBC proportion was equal or greater than the median according to the distribution of each FA in the control group; while they were assigned a score of 0 if the proportion was below the median (the higher status, the higher score). For FAs presumed to be detrimental (C16:0; C18:0; C16:1n-7), individuals whose RBC proportion was below the median according to the distribution in the control group were assigned a value of 1, and individuals whose RBC proportion was at or above the median were assigned a value of 0 (the lower status, the better score). Cut-offs for the 8 items are reported in Supplemental Table 2. Finally, the blood-based LFQ score was calculated for each participant by summing the scores of the 8 components, higher scores indicating higher lipidomic fat quality (minimum, 0; maximum, 8). We performed identical procedures to calculate the LFQ score of the participants from the Framingham Offspring Study cohort.

### Statistical analyses

Categorical variables are expressed as percentages and continuous variables are expressed as means and standard deviation (SD). Normal distribution of continuous variables was assessed with normal Q-Q plots, and skewed (non-normal) variables were rank-transformed.

In EPIC-Spain, we used the chi-square test and the one-way ANOVA test to search for differences between cases and controls for several demographic, clinical and dietary variables of the study population at baseline, as well as blood LFQ score. We next examined the association between blood LFQ score and the risk of ischemic stroke using logistic regression analyses. The LFQ score was analyzed as both continuous and in categories (i.e. tertiles), with scores grouped into three ranks (0–3, 4, and 5–8 points), using the lowest category (0–3) as the reference group. We report results as odds ratios (OR) with corresponding 95% confidence intervals (CI). In all cases, we constructed three models: Model 1 was adjusted for variables used for matching, namely recruiting center (Granada, Murcia, and Navarra), sex, age at baseline, and date of extraction; Model 2 also included baseline variables related to cardiovascular risk, namely body mass index, prevalent hypertension (y/n), prevalent diabetes (y/n), and smoking status (never/former/current); finally, Model 3 was further adjusted for estimated baseline consumption of fruits and vegetables, red meat, fiber, and alcohol.

In the Framingham Offspring Study cohort, associations between the LFQ score (treated both as a continuous and as a categorical variable [0–3; 4; 5–8]) and risk of incident ischemic stroke were examined using multivariable Cox proportional hazards models. Schoenfeld residuals were used to confirm the proportional hazards assumption. Results are reported as hazard ratios (HR) with corresponding 95% CIs. Model 1 was adjusted for sex and age at baseline; Model 2 also adjusted models for body mass index, prevalent hypertension (y/n), prevalent diabetes (y/n), and smoking status (current/not); finally, Model 3 was further adjusted for estimated baseline consumption of fruits and vegetables, red meat, fiber, and alcohol. As a sensitivity analysis, we further adjusted for prevalent atrial fibrillation at baseline.

Alternatively, we developed a score using a “non-dichotomous, weighted” approach. To this end, in the EPIC-Spain cohort we first tested whether there was evidence of nonlinearity for each contributor to the LFQ score and assessed the shape of the association using penalized splines with 2 degrees of freedom. We then computed the *Z*-score of the 8 components, and we performed the logistic regression analysis (Model 3) for each item as exposure of interest. We then computed the weighted LFQ score by adding the result of beta value from individual logistic regression analyses × *Z*-score for each contributing item. We finally performed the logistic regression analysis (Models 1, 2, and 3) using the non-dichotomous, weighted blood LFQ score as exposure of interest. We conducted a receiver operating characteristic (ROC) curve analysis to test the accuracy of the LFQ score and the non-dichotomous, weighted blood LFQ score to discriminate for ischemic stroke cases in EPIC-Spain cohort. Similarly, in the Framingham Offspring Study cohort, we also computed the Z-score of the 8 components, we computed the weighted LFQ score by adding the result of beta values in individual logistic regression analyses derived from EPIC-Spain × *Z*-score of each item in Framingham Offspring Study cohort, and we performed the logistic regression analysis (Models 1, 2, and 3) using the non-dichotomous, weighted blood LFQ score as exposure of interest.

Statistical analyses were performed using SPSS 23 (SPSS Inc., Chicago, IL) (EPIC-Spain) and R (version 4.2.0; Framingham Offspring Study cohort). For all analyses, a two-sided *p* < 0.05 was considered significant.

## Results

Table 1 displays baseline characteristics of the cases and controls in EPIC-Spain cohort. The mean age of participants was 55 years, and 46.8% were women. Differences between cases of incident ischemic stroke and matched controls were restricted to cardiovascular risk factors, with baseline body mass index, prevalence of diabetes, and prevalence of hypertension being significantly higher in cases. Supplemental Table 1 reports data on the 22 determined RBC FAs in the EPIC-Spain cohort. Table 2 shows the baseline characteristics of the participants included from the Framingham Offspring Study cohort.

**Table 1.** Baseline characteristics of the EPIC-Spain cohort participants by cases of ischemic stroke and matched controls.

Variable	Cases *n* = 438	Controls *n* = 438	*p*
Age at baseline, y	55.4 ± 7.2	55.2 ± 7.2	0.677
Female sex	205 (46.8)	205 (46.8)	>0.999
Center			>0.999
Granada	57 (13.0)	57 (13.0)	
Murcia	145 (33.1)	145 (33.1)	
Navarra	236 (53.9)	236 (53.9)	
Body mass index, kg/m^2^	29.8 ± 4.1	28.9 ± 3.8	0.001
Diabetes	63 (14.4)	26 (5.9)	<0.001
Hypertension	191 (43.6)	127 (29.0)	<0.001
Smoking status			0.020
Never	236 (53.9)	254 (58.0)	
Current	67 (15.3)	84 (19.2)	
Former	135 (30.8)	100 (22.8)	
Physical activity			0.660
Inactive	174 (39.7)	158 (36.1)	
Moderately inactive	133 (30.4)	144 (32.9)	
Moderately active	87 (19.9)	86 (19.6)	
Active	44 (10.0)	50 (11.4)	
Education			0.222
Primary school	331 (75.6)	321 (73.3)	
High school	43 (9.8)	53 (12.1)	
College	22 (5.0)	32 (7.3)	
Unknown	42 (9.6)	32 (7.3)	
Self-reported dietary data			
Fruits and vegetables, g/d	591 (424; 795)	553 (415; 741)	0.292
Red meat, g/d	37 (13; 64)	32 (15; 57)	0.164
Dairy products, g/d	235 (115; 357)	248 (167; 349)	0.061
Fiber, g/d	25 (20; 31)	24 (20; 31)	0.600
Alcohol intake, g/d	5 (0; 28)	6 (0; 28)	0.511

Values are presented as *n* (%), except for age, body mass index (expressed as mean ± standard deviation), and dietary variables (expressed as median (interquartile ranges)).

*p* between cases and matched controls, obtained by the chi-square test (categorical variables) or by one-way ANOVA (dietary data were rank-transformed), as appropriate.

**Table 2.** Baseline characteristics of the Framingham Offspring Study participants (*n* = 2468).

Variable	Value
Age at baseline, y	66.0 ± 8.8
Female sex	1362 (55.2)
White	2421 (98.1)
Body mass index, kg/m^2^	28.2 ± 5.3
Diabetes	270 (10.9)
Antihypertensive medication use, n (%)	1181 (47.9)
Current smoking	186 (7.5)
Physical activity, hours per day	
Slight activity	5.3 ± 2.5
Moderate activity	3.7 ± 2.2
Heavy activity	0.7 ± 1.2
Education	
No high school degree	18 (0.7)
High school degree	721 (29.2)
More than high school	1714 (69.4)
Unknown	15 (0.6)
Self-reported dietary data	
Fruits and vegetables, servings/week	29.2 (19.2; 41.7)
Red meat, servings/week	4.4 (2.4; 6.5)
Dairy products, servings/week	17.4 (10.9; 26.9)
Fiber, g/d	17.8 (13.3; 23.6)
Alcohol intake, drinks/week	2.0 (0.0; 7.0)
RBC metrics used to compute the LFQ score, % of total fatty acids	
C15:0 + C17:0	0.5 ± 0.1
C18:2n-6	11.0 ± 1.7
C18:3n-3	0.2 ± 0.1
C20:5n-3	0.7 ± 0.5
C22:6n-3	4.8 ± 1.3
C16:0	21.0 ± 1.2
C16:1n-7	0.4 ± 0.2
C18:0	17.9 ± 0.9

Values are presented as either *n* (%), mean ± standard deviation, or median interquartile ranges).

In EPIC-Spain cohort, there were statistical differences for blood LFQ score between cases (mean ± standard deviation, 3.68 ± 1.46; median [interquartile range], 4.00 [3.00–5.00]) and controls (4.00 ± 1.47; median [interquartile range], 4.00 [3.00–5.00]) (*p* = 0.001). Blood LFQ score was associated with lower odds of ischemic risk score when assessed as both continuous variable and per categories (Table 3). Statistically significant lower odds were observed in both analyses for all different models. When explored as continuous variable, in the fully adjusted model, the odds of ischemic stroke were 14% lower (OR: 0.86; 95% CI: 0.77–0.95; *p* = 0.005) for each increase in 1 unit of the LFQ score. Findings were similar when comparing extreme categories of the LFQ score. A fully adjusted model showed that, compared to participants in the lowest category (score 0–3), those in the upper category (score 5–8) showed a statistically significant reduction by 36% of odds of ischemic stroke (OR: 0.64, 95% CI: 0.44–0.94, *p* = 0.021). Findings were also observed in the Framingham Offspring Study cohort (Table 4). Of note, in fully adjusted multivariable Cox proportional hazards models, compared to participants in the lowest LFQ category (score 0–3), those with higher lipidomic fat quality (score 5–8) had approximately half the risk of incident ischemic stroke (HR: 0.49; 95% CI: 0.29–0.84, *p* = 0.020). The association remained exactly the same after further adjustment for prevalent atrial fibrillation at baseline.

**Table 3.** Risk of ischemic stroke for blood lipidomic fat quality (LFQ) score in EPIC-Spain.

Model	OR (95% CI), per 1 unit increase	OR (95% CI) for blood LFQ score categories			
		Score from 0 to 3 *n* = 229	Score = 4 *n* = 337	Score from 5 to 8 *n* = 310	*p* Trend value
	438 cases	127 cases	175 cases	136 cases	
Model 1	0.85 (0.78–0.94)	1.00 (Ref)	0.87 (0.61–1.22)	0.62 (0.44–0.87)	0.016
Model 2	0.87 (0.79–0.96)	1.00 (Ref)	0.85 (0.59–1.21)	0.66 (0.46–0.95)	0.076
Model 3	0.86 (0.77–0.95)	1.00 (Ref)	0.83 (0.58–1.19)	0.64 (0.44–0.94)	0.064

CI: confidence interval; OR: odds ratio.

Data obtained by logistic regression analysis. Model 1, adjusted for recruiting center (Granada, Murcia, and Navarra), sex, age at baseline, and date of extraction; Model 2, + adjusted for baseline body mass index, prevalent hypertension (y/*n*), prevalent diabetes (y/*n*), and smoking status (never/former/current); Model 3, + adjusted for baseline estimated consumption of fruits and vegetables, red meat, as well as fiber and alcohol intake.

*p*-Value computed by assigning scores of 0, 1, and 2 to the three blood LFQ score categories in the logistic regression model.

**Table 4.** Risk of ischemic stroke for blood lipidomic fat quality (LFQ) score in Framingham Offspring Study.

Model	HR (95% CI), per 1 unit increase	HR (95% CI) for blood LFQ score categories			
		Score from 0 to 3 *n* = 1027	Score = 4 *n* = 781	Score from 5 to 8 *n* = 660	*p* for trend
	121 cases	58 cases	44 cases	19 cases	
Model 1	0.83 (0.70–0.97)	1.00 (Ref)	1.03 (0.70–1.53)	0.47 (0.28–0.79)	0.010
Model 2	0.84 (0.72–0.99)	1.00 (Ref)	1.05 (0.71–1.56)	0.50 (0.30–0.85)	0.022
Model 3	0.83 (0.70–0.99)	1.00 (Ref)	1.04 (0.70–1.56)	0.49 (0.29–0.84)	0.020

CI: confidence interval; HR: hazard ratio.

Data obtained from a Cox-proportional hazards model. Model 1, adjusted for sex and age at baseline; Model 2, + adjusted for baseline body mass index, prevalent hypertension (y/n), prevalent diabetes (y/n), and smoking status (current/not); Model 3, + adjusted for baseline estimated consumption of fruits and vegetables, red meat, and alcohol, as well as fiber intake.

*p*-Value computed by assigning scores of 0, 1, and 2 to the three blood LFQ score categories in the Cox-model.

We further examined the associations for non-dichotomous, weighted LFQ score. We searched for non-linear associations, creating a penalized spline curve of the logarithm of OR of ischemic stroke in relation to each contributing item. No evidence of non-linearity was observed (Supplemental Figure 2). When examining the association for each item on its own, statistical significance was only observed for RBC palmitic acid (C16:0) (Supplemental Table 3). We observed statistical differences for the non-dichotomous, weighted blood LFQ score between cases (mean ± standard deviation, −0.0238 ± 0.18053) and controls (0.0238 ± 0.18678; *p* < 0.001). In the fully adjusted model, the odds of ischemic stroke were 71% lower (OR: 0.29; 95% CI: 0.13–0.65; *p* = 0.003) for each increase in 1 unit of the non-dichotomous, weighted LFQ score (EPIC-Spain cohort; Supplemental Table 4). Areas under the curve (AUC) for ROC curves were essentially the same using either LFQ score or the non-dichotomous, weighted LFQ score (Supplemental Figure 3). In the Framingham Offspring Study cohort, we observed an inverse association between non-dichotomous, weighted LFQ score and ischemic stroke in the verge of statistical significance (HR: 0.62; 95% CI: 0.38–1.02; *p* = 0.058 for each increase in 1 unit; Supplemental Table 5).

## Discussion

In this case-control study nested within a prospective cohort of a large general population sample from Spain, we performed lipidomics in blood samples from 438 cases of ischemic stroke and 438 matched controls, developed a blood lipidomic score based on a panel of pre-specified RBC FAs that can be modified through dietary changes, and found that increasing the LFQ score (reflecting better fat quality) related to a lower risk of ischemic stroke. The external validity of our findings is attested by the inverse associations also observed in an independent, well-reputed, community-based cohort of participants with a different environmental background. Besides reinforcing the link between diet and ischemic stroke by using objective biomarkers of dietary intake, we provide a preliminary tool which might be useful for identification, prevention (i.e. dietary changes), and eventually monitoring of populations at increased risk of ischemic stroke.

Several aspects of our work deserve to be underlined. First, rather than focusing on an individual FA or FA family (“single-nutrient approach”), we created a score that combines different diet-related FAs, hence better capturing the complexity of real-world dietary intake (nutrient-nutrient interactions, intercorrelations, and food matrix characteristics). To this end, we included any FA the blood status of which can be used as a biomarker of diet. We deem the pre-defined selection of items (and the presumed beneficial/detrimental effect or each item) as optimal, as attested by the found robust, independent, statistically significant associations for the LFQ score, according to the hypothesis of higher LFQ, lower odds of ischemic stroke. In addition, we tailored the LFQ score, in the sense that it goes beyond the over-simplistic “saturated FAs are bad, polyunsaturated FAs are good,” which has been a common dogma in the diet-heart hypothesis. In this regard, our score considers that saturated FAs can be either beneficial (C15:0, C17:0, as supplied by dairy) or detrimental (C16:0, C18:0, as end-product of overfeeding with carbohydrates).

Second, we used objective biomarkers of dietary intake as opposed to self-reported dietary data. Despite being extensively validated, methods that require participants to recall and record their food and beverage consumption over a fixed period of time (i.e. 24-h recalls or food frequency questionnaires) have a number of limitations that affect both the accuracy and precision of the measurement (i.e. interviewer bias, incorrect portion size estimations, coding and computational errors, and the need of updated and comprehensive nutritional databases). Blood-based biomarkers circumvent such limitations.^5,25^ Of note, we worked with RBC metrics. Given the RBC lifespan of approximately 120 days, RBC FAs are not as labile as plasma FAs. Therefore, the RBC FA pattern provides a stable indicator of the nutritional environment over the course of several months, being analogous to hemoglobin A1c as the preferred metric for a time-averaged marker of blood glucose status (vs plasma glucose).

We finally would like to underscore the clinical relevance of the findings. Pending confirmation of its applicability in other populations, this score is intended to be a simple, early-warning index that will allow for targeted intervention of individuals at high-risk of stroke, advising for simple and personalized changes (i.e. increase the consumption of fatty fish, consider use of fish oils, increase the consumption of nuts and oily seeds, increase the consumption of dairy, and/or limit the intake of refined sugars). For the sake of simplicity, the first tentative cut-off was created using medians. Despite being aware of the downsides of using dichotomous values,^26^ we ultimately hope to generate a tool that, by rapidly informing about which aspects of the diet should be improved, can help to limit the prevalence and societal burden of this disease. However, the instrumental variable here reported is not a prognostic score, whose evaluation would require further metrics and analyses which are beyond the scope of this paper. In addition, further research is needed to explore whether this LFQ score may effectively predict subtypes of ischemic stroke, hemorrhagic stroke, its severity, or even the prognosis after the event.

Our study has several limitations. First, due to its observational nature, we cannot infer causality between the blood LFQ score and incident ischemic stroke. Uncaptured factors might have contributed to the observed associations. However, the thorough characterization of study participants for cardiovascular risk factors and lifestyle allowed us the inclusion of many confounders in multivariable models, including foods and nutrients long related to the risk of ischemic stroke (fruits and vegetables, red meat, fiber, and alcohol). Second, the EPIC Spanish cohort is not a random representative sample of the population from which they were selected, and therefore dietary patterns and healthcare access may not be fully representative of broader populations. However, the findings were also observed in a cohort with a different background. Third, our samples are mostly composed of European descent, which limits the generalization of our results to other ethnic groups. Fourth, we pre-defined FAs to be included into the score and assigned the “0” or “1” score according to the median value of the control group, but we did no formal assessment of how the pre-determined metric could be further optimized through statistical selection of the FAs to include and how to weight them. However, the most predominant aggregation technique to establish diet-related scores is by far linear aggregation with equally weighted indicators.^27–29^ In addition, when using a LFQ score computed using a non-dichotomous and weighting approach, results remained essentially the same. Finally, associations are based on measurement of RBC FAs at baseline, which do not account for trends or changes over time. However, in the context of the Physicians’ Health Study and incident heart failure, a correlation between measurements of circulating diet-related FAs spaced by 15 years was reported, thus validating the usefulness of a single measurement of FAs in estimating the risk of incident disease over long-term follow-up.^30^

In conclusion, using data from 438 cases of ischemic stroke and 438 matched controls from EPIC-Spain cohort, we developed a blood-based LFQ score that includes pre-defined metrics that can be modified by diet. We found that individuals with a low blood-based LFQ score have higher odds of ischemic stroke (score 0–3, 44% higher risk as compared to score 5–8). Such findings were also observed in an independent cohort of participants with a different environmental background. In the era of personalized nutrition, our results suggest that increasing the blood-based LFQ of the target population might be an easy and safe strategy in the primary prevention of ischemic stroke.

## Supplementary Material

sj-docx-1-eso_23969873251367250

## Data Availability

Derived data that support the findings of this study are available from the corresponding authors on reasonable request. Raw data are not available because of reasons of sensitivity.

## Correction (October 2025):

The abstract has been updated to include additional text in the Patients and methods section. See Article Note for further details.

## Article Note

The abstract was updated to add the following text within the Patients and methods section:

“<the median of the control group. LFQ score resulted from the 8-component sum (range=0-8; higher values, higher fat quality). We explored the validity of findings in a different background”.
