# Supplementary material for: Development of a blood-based lipidomic fat quality score for the risk of ischemic stroke
Source: Eur Stroke J. 2026 Jan 1;11(1):23969873251367250. doi: 10.1093/esj/23969873251367250 (PMC12866280; doi:10.1093/esj/23969873251367250)
Supplement: sj-docx-1-eso_23969873251367250 [file sj-docx-1-eso_23969873251367250.docx]

Supplementary material for “**Development of a blood-based lipidomic fat quality score for the risk of ischemic stroke**”, by Lázaro et al.

**Supplementary Figure 1.** Flow chart of the study

**
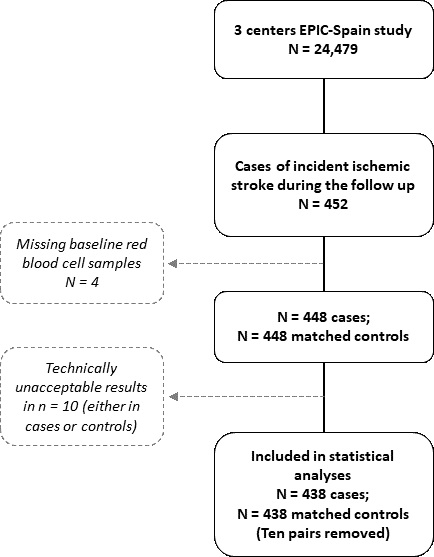
**

**Supplementary Figure 2.** Penalized spline plots for the risk of ischemic stroke for each of contributing items to LFQ score


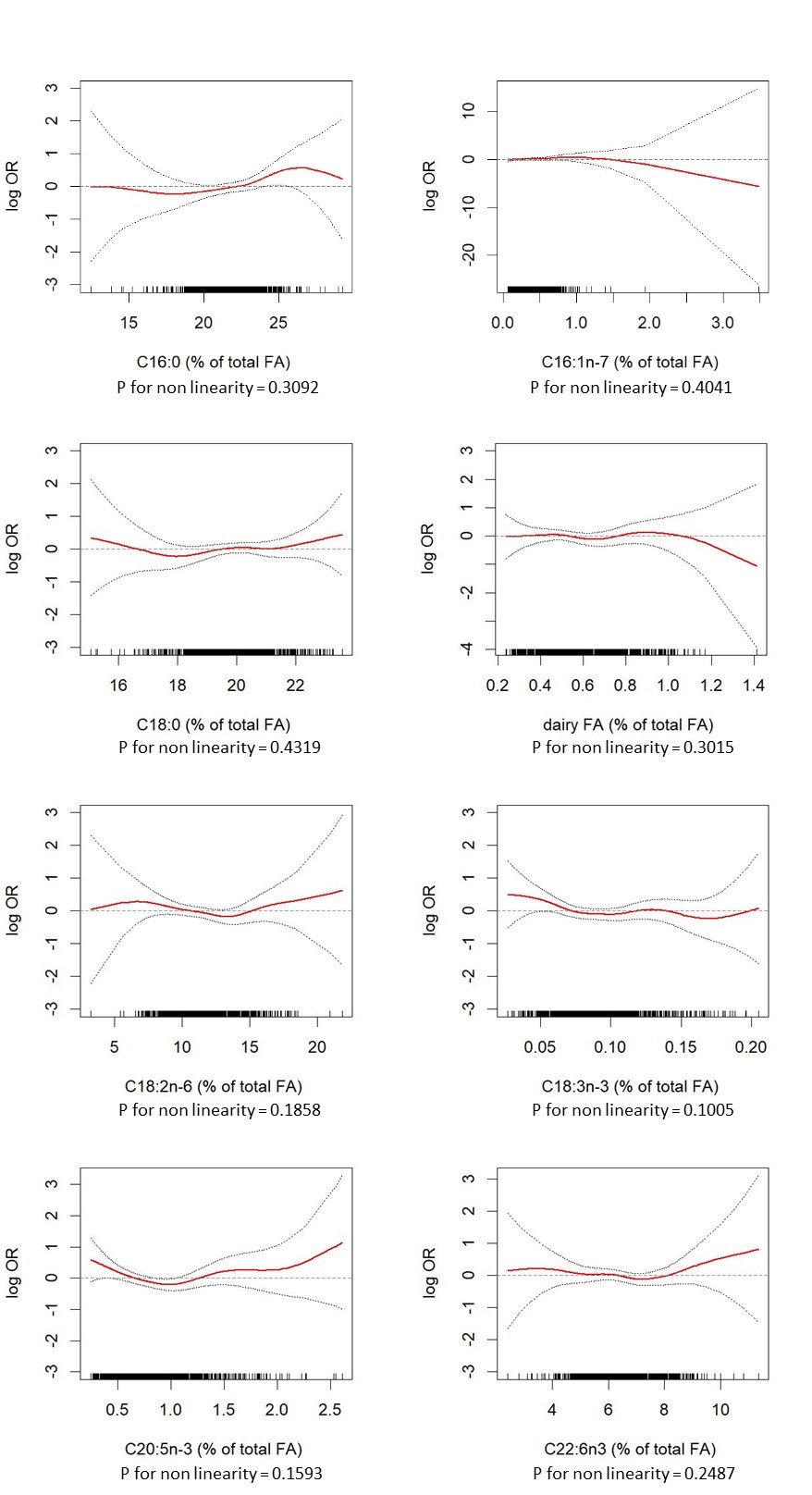


Solid red line represents the logarithm of OR, and dashed lines depict the 95% confidence intervals.

**Supplementary Figure 3.** Receiver operating characteristic (ROC) curve to discriminate between non-weighted and non-dichotomous, weighted scores


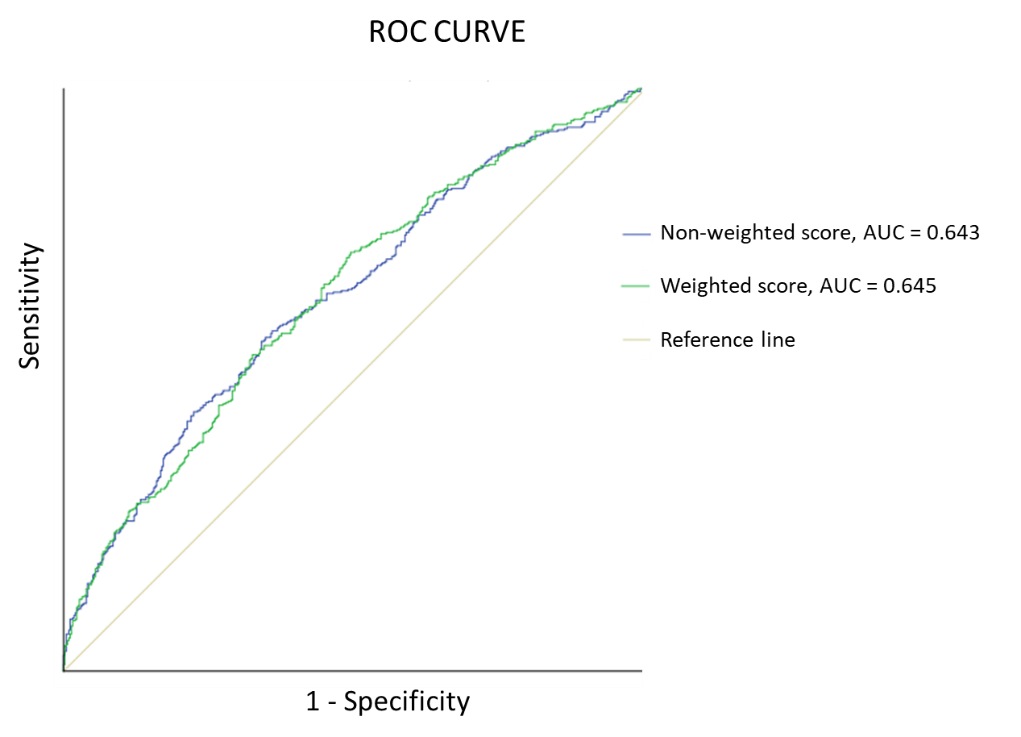


**Supplementary Table 1**. Baseline red blood cell fatty acid proportions of the study population by cases of ischemic stroke and matched controls

| **Fatty acid** | **Cases**  **n = 438** | **Controls**  **n = 438** |
| --- | --- | --- |
| C12:0 | 0.02 (0.02 to 0.02) | 0.02 (0.02 to 0.02) |
| C14:0 | 0.55 (0.52 to 0.58) | 0.53 (0.50 to 056) |
| C15:0 | 0.14 (0.14 to 0.15) | 0.14 (0.14 to 0.15) |
| C16:0 | 21.89 (21.71 to 22.07) | 21.50 (21.33 to 21.68) |
| C16:1n-7 | 0.31 (0.29 to 0.33) | 0.30 (0.27 to 0.32) |
| C17:0 | 0.43 (0.42 to 0.45) | 0.44 (0.42 to 0.45) |
| C18:0 | 19.94 (19.82 to 20.06) | 19.86 (19.75 to 19.98) |
| C18:1n-9 *cis* | 15.32 (15.07 to 15.57) | 15.78 (15.53 to 16.06) |
| All-C18:1 *trans* | 0.35 (0.33 to 0.38) | 0.38 (0.36 to 0.41) |
| C18:2n-6 | 11.38 (11.16 to 11.60) | 11.52 (11.31 to 11.73) |
| C18:3n-3 | 0.09 (0.09 to 0.09) | 0.09 (0.09 to 0.10) |
| C20:0 | 0.11 (0.10 to 0.11) | 0.11 (0.10 to 0.11) |
| C20:1n-9 | 0.29 (0.28 to 0.30) | 0.30 (0.29 to 0.31) |
| C20:2n-6 | 0.36 (0.35 to 0.37) | 0.36 (0.34 to 0.37) |
| C20:3n-6 | 1.43 (1.39 to 1.46) | 1.39 (1.35 to 1.42) |
| C20:4n-6 | 18.90 (18.70 to 19.11) | 18.77 (18.57 to 18.98) |
| C22:0 | 0.10 (0.10 to 0.11) | 0.11 (0.10 to 0.11) |
| C22:1n-9 | 0.39 (0.36 to 0.42) | 0.42 (0.38 to 0.46) |
| C20:5n-3 | 0.88 (0.84 to 0.91) | 0.87 (0.84 to 0.90) |
| C24:0 | 0.28 (0.27 to 0.28) | 0.28 (0.27 to 0.29) |
| C24:1n-9 | 0.30 (0.29 to 0.31) | 0.31 (0.30 to 0.32) |
| C22:6n-3 | 6.52 (6.41 to 6.64) | 6.52 (6.41 to 6.63) |

Data are expressed as means (95% confidence interval).

P between cases and matched controls obtained by 1-way ANOVA.

**Supplementary Table 2**. Cut-off values to compute the blood-based lipidomic fat quality score

|  | **Cut-off (median according to the distribution in the control group)*** | **Scoring** |
| --- | --- | --- |
| Beneficial fatty acids |  |  |
| C15:0 + C17:0 | 0.5446 | 1 if RBC proportion is equal or greater |
| C18:2n-6 | 11.38735 | 1 if RBC proportion is equal or greater |
| C18:3n-3 | 0.08982 | 1 if RBC proportion is equal or greater |
| C20:5n-3 | 0.82721 | 1 if RBC proportion is equal or greater |
| C22:6n-3 | 6.57526 | 1 if RBC proportion is equal or greater |
| Detrimental fatty acids |  |  |
| C16:0 | 21.52371 | 1 if RBC proportion is below |
| C16:1n-7 | 0.22105 | 1 if RBC proportion is below |
| C18:0 | 19.89266 | 1 if RBC proportion is below |

* Proportion of the total amount of 22 determined fatty acids

**Supplementary Table 3**. Risk of ischemic stroke for isolated items included in the blood lipidomic fat quality score (EPIC-Spain)

| **Score item** | **OR (95% CI)** | **Beta** | **P** |
| --- | --- | --- | --- |
| C15:0 + C17:0 | 1.001 (0.809 to 1.238) | 0.001 | 0.993 |
| C18:2n-6 | 0.953 (0.820 to 1.108) | -0.048 | 0.530 |
| C18:3n-3 | 0.921 (0.800 to 1.061) | -0.082 | 0.253 |
| C20:5n-3 | 0.990 (0.854 to 1.147) | -0.010 | 0.891 |
| C22:6n-3 | 0.983 (0.852 to 1.135) | -0.017 | 0.819 |
| C16:0 | 1.172 (1.013 to 1.357) | 0.159 | 0.033 |
| C16:1n-7 | 1.031 (0.866 to 1.228) | 0.030 | 0.733 |
| C18:0 | 1.076 (0.932 to 1.241) | 0.073 | 0.319 |

OR, odds ratio; CI, confidence interval.

Data obtained by logistic regression analysis (one separate model for each item, using Z-score values as exposure of interest), adjusted for recruiting center (Granada / Murcia / Navarra), sex, age at baseline, date of extraction, baseline body mass index, prevalent hypertension (y / n), prevalent diabetes (y / n), smoking status (never / former / current), baseline estimated consumption of fruits and vegetables, red meat, as well as fiber and alcohol intake.

**Supplementary Table 4**. Risk of ischemic stroke for non-dichotomous, weighted blood lipidomic fat quality (LFQ) score in EPIC-Spain

|  | **OR (95% CI),**  **per 1-unit increase** | **P** |
| --- | --- | --- |
| Model 1 | 0.230 (0.108 to 0.489) | <0.001 |
| Model 2 | 0.298 (0.138 to 0.647) | 0.002 |
| Model 3 | 0.287 (0.128 to 0.646) | 0.003 |

OR, odds ratio; CI, confidence interval.

The weighted score was calculated using inverse betas from logistic regression of isolated endpoints (the higher the score, the better fat quality), using the following formula:

Weighted score = Zscore C16:0 * -0.159 + Zscore C16:1 * -0.030 + Zscore C18:0 * -0.073 + Zscore C18:2n6 * 0.048 + Zscore C18:3n3 * 0.082 + Zscore C20:5n3 * 0.010 + Zscore C22:6n3 * 0.017 + Zscore sum of C15:0 and C17:0 * 0.001.

Data obtained by logistic regression analysis. Model 1, adjusted for recruiting center (Granada, Murcia, and Navarra), sex, age at baseline, and date of extraction; Model 2, + adjusted for baseline body mass index, prevalent hypertension (y/n), prevalent diabetes (y/n), and smoking status (never/former/current); Model 3, + adjusted for baseline estimated consumption of fruits and vegetables, red meat, as well as fiber and alcohol intake.

**Supplementary Table 5**. Risk of ischemic stroke for non-dichotomous, weighted blood lipidomic fat quality (LFQ) score in Framingham Offspring Study cohort

|  | **HR (95% CI),**  **per 1-unit increase** | **P** |
| --- | --- | --- |
| Model 1 | 0.69 (0.45 to 1.05) | 0.086 |
| Model 2 | 0.72 (0.47 to 1.12) | 0.143 |
| Model 3 | 0.62 (0.38 to 1.02) | 0.058 |

HR, hazard ratio; CI, confidence interval.

The weighted score was calculated using inverse betas from logistic regression of isolated endpoints (the higher the score, the better fat quality), using the following formula:

Weighted score = Zscore C16:0 * -0.159 + Zscore C16:1 * -0.030 + Zscore C18:0 * -0.073 + Zscore C18:2n6 * 0.048 + Zscore C18:3n3 * 0.082 + Zscore C20:5n3 * 0.010 + Zscore C22:6n3 * 0.017 + Zscore sum of C15:0 and C17:0 * 0.001.

Data obtained from a Cox-proportional hazards model. Model 1, adjusted for sex and age at baseline; Model 2, + adjusted for baseline body mass index, prevalent hypertension (y/n), prevalent diabetes (y/n), and smoking status (current/not); Model 3, + adjusted for baseline estimated consumption of fruits and vegetables, red meat, and alcohol, as well as fiber intake.
